# Identification of proteins potentially associated with renal aging in rats

**DOI:** 10.18632/aging.101460

**Published:** 2018-06-14

**Authors:** Diangeng Li, Delong Zhao, Weiguang Zhang, Qian Ma, Dong Liu, Qi Huang, Ying Zheng, Xueyuan Bai, Xuefeng Sun, Xiangmei Chen

**Affiliations:** 1 Department of Nephrology, Chinese PLA General Hospital, Chinese PLA Institute of Nephrology, State Key Laboratory of Kidney Diseases, National Clinical Research Center of Kidney Diseases, Beijing 100853, China

**Keywords:** renal aging, kidney transplant, proteomic, bioinformatics analysis, SOD1

## Abstract

We established a young (Y)-old (O) rat kidney transplantation model. With this model, we detected no age-related differences in renal structure between Y→Y and Y→O kidneys or O→O and O→Y kidneys. However, we did detect differences in levels of the senescence markers β-gal and p16 as well as the inflammatory cytokines TNF-α and IL-1β. Using proteomics analysis we detected 66 proteins associated with suppression of aging and 73 proteins associated with enhancement of aging. After construction of a protein-protein interaction network, a total of 73 nodes and 99 edges were analyzed using MCODE, and three significant modules were selected. GO and KEGG analyses showed that these proteins were mainly located in mitochondria and were largely related to oxidative stress. Among them, SOD1 expression was lower in Y→O than Y→Y kidneys and higher in O→Y than O→O kidneys. Acetylated (Ac)-NF-κB showed the opposite expression profile. In addition, SOD1 expression was higher in primary tubular epithelial cells from young rats than old rats, and SOD1 knockdown led to increased Ac-NF-κB expression. These findings suggest the local renal environment, particularly oxidative stress/mitochondrial function, affects renal aging.

## Introduction

Thanks to advances in science and medicine people are living longer and aging of the population has become an important issue. By 2030, the segment of the population over 65 years will have nearly doubled, and the incidence of age-associated diseases is expected to increase in parallel [1]. As older age is associated with the risk of kidney disease, chronic loss of kidney function is of significant importance. Kidney disease not only impairs patients’ quality of life and is a potential burden to families and society, it is also a major independent risk factor for cardiovascular morbidity and mortality [2]. At present, however, the molecular basis of renal aging remains unclear.

The process of renal aging involves multiple factors. We therefore established a novel young-old rat kidney transplant model to explore ways to prevent renal aging through direct examination of the kidney within its environment. With this method, we are able to observe the impact of the local renal environment (e.g., circulation, body fluids, etc.) on renal aging and to screen key factors/pathways affecting renal aging. Previous studies showed that young-old donor kidney transplantation is feasible [3] and that the graft survival rate for recipients of old donor kidneys equals that for standard deceased donor kidney transplants. Moreover, donor age appears to have no significant impact on the survival of the graft or the patients [4]. Although the experimental technique of rat kidney transplantation is now well established, there are certain technical difficulties that must be overcome to establish a young-old rat kidney transplant model, which is a major highlight of this study. The findings of this study could potentially provide new direction for delaying renal aging.

## RESULTS

### Establishing a young - old rat kidney transplant model

As shown in Table 1, following kidney transplantation, perioperative and postoperative mortality increased stepwise from the Y→Y to the O→O group. Nonetheless, this rat renal allograft transplantation model had fewer complications and an improved survival rate, thereby providing a more practical and reliable model for further experiments.

**Table 1:** Survival details of Young - Old rats kidney transplanted model establishment.

**Group**	**Perioperative Mortality % (n/n)**		**Mortality 16 weeks after surgery** **% (n/n)**	**Survival rate** ** (%)**
	**Intranperative period**	**24 hours after the surgery**		
Y con	-	-	-	100
Y→Y	14.29 (1/7)	-	14.29 (1/7)	85.71
Y→O	11.11 (1/9)	22.22 (2/9)	33.33 (3/9)	66.67
O→Y	25.00 (2/8)	-	25.00 (2/8)	75.00
O→O	36.36 (4/11)	18.18 (2/11)	54.55 (6/11)	45.45
O con	-	-	-	100

**Figure 1:**
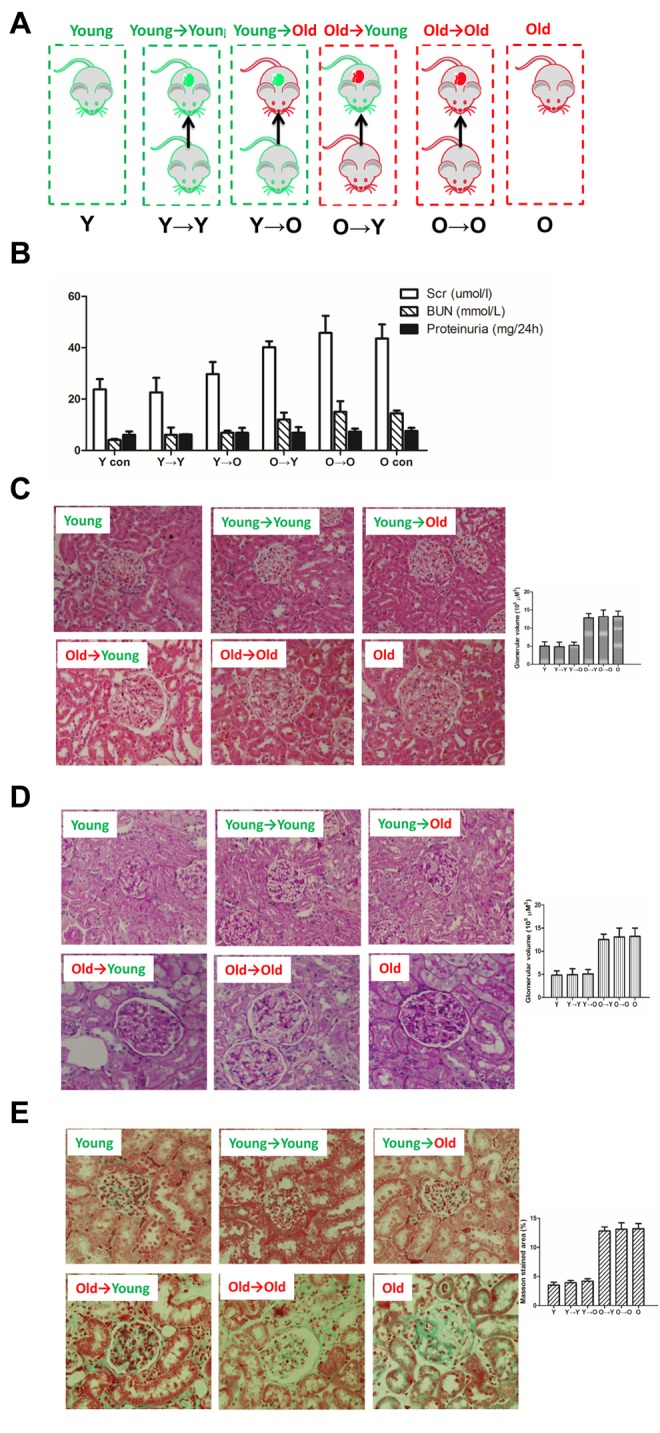
**The histological examinations result from each group in this study.** (**A**) Schematic diagram of kidney transplantation. Y→Y, young rat with kidney transplanted from young rat (young control); Y→O, old rat with kidney transplanted from young rat; O→Y, young rat with kidney transplanted from old rat; O→O, old rat with kidney transplanted from old rat (old control). (**B**) Renal functionality (serum creatinine, serum BUN, proteinurine) in each group. (**C**) HE staining of kidney tissue samples from each group (×200). **(D**) PAS staining of kidney tissue samples from each group (×200). (**E**) Masson staining of kidney tissue samples from each group (×200);

As shown in Figure 1B, there was no significant difference in renal function between the O control (O→O) and O→Y rats or between the Y control (Y→Y) and Y→O rats.

### Histological examination

Histological examination was performed using hematoxylin and eosin (HE), periodic acid Schiff (PAS) and Masson staining (Figure 1C-E). HE staining revealed the morphological changes within the kidney tissue. Aged kidneys exhibited increased glomerular volume and swelling of the tubular epithelium. PAS staining revealed marked glycogen deposition in the aged kidneys, indicating tissue damage. Masson staining was used to assess renal fibrosis. In each case, there was no significant difference between the kidneys used for O→O and O→Y transplantation or those used for Y→Y and Y→O transplantation

### Senescence-associated β-galactosidase staining and p16 expression

**Figure 2:**
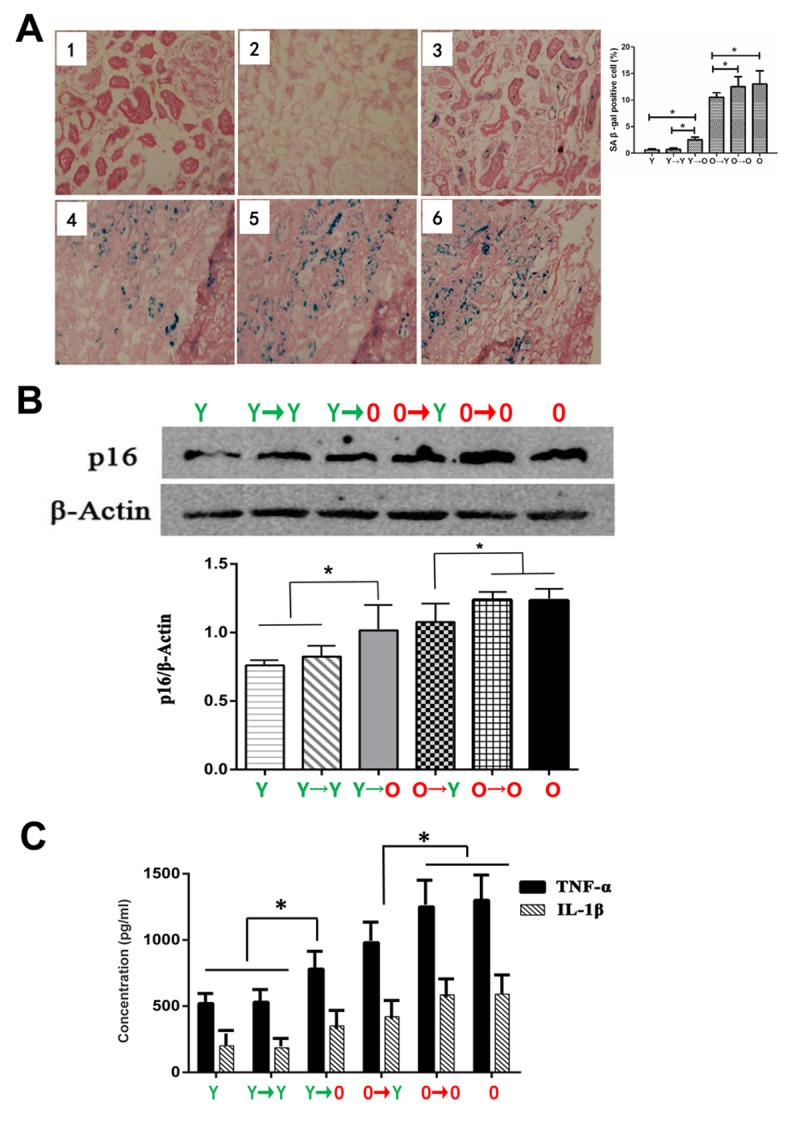
**Levels of aging biomarkers.** (**A**) SA-β-gal staining in each group (*p < 0.05). (**B**) Western blot showing p16 expression levels (*p < 0.05). (**C**) Results of specific ELISAs showing expression levels of TNF-α and IL-1β (*p < 0.05).

Senescent cells are characterized by growth arrest, enlarged and flat cellular morphology, and an expression profile characterized by expression of senescence-associated genes. The most commonly used marker to identify senescent cells is β-gal activity. As shown in Figure 2A, levels of β-gal expression were lower in the O→Y transplanted kidney tissue than in O→O transplanted kidneys. Conversely, β-gal expression was higher in Y→O transplanted kidney tissue than in Y→Y transplanted kidneys. A similar profile was seen with p16, another biomarker of senescence (Figure 2B).

### Inflammatory cytokine expression

Because renal aging is associated with chronic inflammation, we assessed expression of two inflammatory cytokines, TNF-α and IL-1β, in the four groups. We found that levels of TNF-α and IL-1β were higher in the Y→O than the Y→Y group, and were lower in the O→Y than the O→O group (Figure 2C).

### Identification and quantification of differentially expressed proteins

Compared with the Y→Y group, we detected 391 differentially expressed proteins (181 upregulated proteins, 210 downregulated proteins) in the Y→O group. Compared with the O→O group, we detected 380 differentially expressed proteins (169 upregulated proteins, 211 downregulated proteins) in the O→Y. Sixty-six proteins were associated with anti-aging (downregulated in the Y→O *vs.* Y→Y and upregulated in the O→Y *vs.* O→O), and 73 proteins were associated with pro-aging (up-regulated in the Y→O *vs.* Y→Y and downregulated in the O→Y *vs.* O→O).

### Gene Ontology (GO) term enrichment analysis

We uploaded all differentially expressed proteins (DEPs) to the online software DAVID to identify overrepresented GO categories and Kyoto Encyclopedia of Genes and Genomes (KEGG) pathways. The GO analysis showed that DEPs were significantly enriched in biological processes, including cellular iron ion homeostasis, lipid metabolic process, and negative regulation of cholesterol biosynthetic process. For cell components, the DEPs were enriched in extracellular exosome, extracellular space, mitochondrion, and others. In addition, a GO molecular function analysis showed that the DEPs were significantly enriched for proton-transporting ATP synthase activity, rotational mechanism, poly(A) RNA binding, peptide binding, and protein binding (Table 2).

**Table 2:** Gene ontology analysis of differentially expressed proteins associated with renal aging.

**Category**	**pathway ID/ pathway description**	***P* value**
GOTERM_BP_DIRECT	GO:0006879~cellular iron ion homeostasis	8.0E-4
GOTERM_BP_DIRECT	GO:0006629~lipid metabolic process	
GOTERM_BP_DIRECT	GO:0045541~negative regulation of cholesterol biosynthetic process	5.1E-3
GOTERM_BP_DIRECT	GO:0051055~negative regulation of lipid biosynthetic process	6.1E-3
GOTERM_BP_DIRECT	GO:0044794~positive regulation by host of viral process	7.2E-3
GOTERM_BP_DIRECT	GO:0045471~response to ethanol	1.6E-2
GOTERM_BP_DIRECT	GO:0045780~positive regulation of bone resorption	1.9E-2
GOTERM_BP_DIRECT	GO:0015986~ATP synthesis coupled proton transport	2.4E-2
GOTERM_BP_DIRECT	GO:0055114~oxidation-reduction process	2.7E-2
GOTERM_BP_DIRECT	GO:0060999~positive regulation of dendritic spine development	2.9E-2
GOTERM_BP_DIRECT	GO:0001937~negative regulation of endothelial cell proliferation	3.3E-2
GOTERM_BP_DIRECT	GO:0001895~retina homeostasis	3.5E-2
GOTERM_BP_DIRECT	GO:0006953~acute-phase response	3.8E-2
GOTERM_BP_DIRECT	GO:0007568~aging	4.1E-2
GOTERM_BP_DIRECT	GO:0046034~ATP metabolic process	4.6E-2
GOTERM_BP_DIRECT	GO:0007566~embryo implantation	5.3E-2
GOTERM_BP_DIRECT	GO:0007283~spermatogenesis	5.5E-2
GOTERM_BP_DIRECT	GO:0050727~regulation of inflammatory response	6.0E-2
GOTERM_BP_DIRECT	GO:0034599~cellular response to oxidative stress	7.7E-2
GOTERM_CC_DIRECT	GO:0070062~extracellular exosome	2.1E-11
GOTERM_CC_DIRECT	GO:0005615~extracellular space	3.9E-8
GOTERM_CC_DIRECT	GO:0005739~mitochondrion	3.4E-6
GOTERM_CC_DIRECT	GO:0043209~myelin sheath	2.3E-5
GOTERM_CC_DIRECT	GO:0072562~blood microparticle	1.6E-4
GOTERM_CC_DIRECT	GO:0031012~extracellular matrix	1.5E-3
GOTERM_CC_DIRECT	GO:0005576~extracellular region	3.5E-3
GOTERM_CC_DIRECT	GO:0005623~cell	5.2E-3
GOTERM_CC_DIRECT	GO:0031232~extrinsic component of external side of plasma membrane	7.3E-3
GOTERM_CC_DIRECT	GO:0005753~mitochondrial proton-transporting ATP synthase complex	2.0E-2
GOTERM_CC_DIRECT	GO:0005764~lysosome	2.1E-2
GOTERM_CC_DIRECT	GO:0006743~mitochondrial inner membrane	3.3E-2
GOTERM_CC_DIRECT	GO:0016020~membrane	4.3E-2
GOTERM_MF_DIRECT	GO:0046933~proton-transporting ATP synthase activity, rotational mechanism	1.9E-2
GOTERM_MF_DIRECT	GO:0044822~poly(A) RNA binding	3.0E-2
GOTERM_MF_DIRECT	GO:0042277~peptide binding	8.2E-2
GOTERM_MF_DIRECT	GO:0005515~protein binding	8.6E-2

### KEGG pathway analysis

Table 3 contains the most significantly enriched KEGG pathways of the analyzed DEPs. The DEPs were enriched in metabolic pathways, Huntington's disease, carbon metabolism, Alzheimer's disease, propanoate metabolism, pyruvate metabolism, and glutathione metabolism.

**Table 3:** KEGG pathway analysis of differentially expressed proteins associated with renal aging.

**Pathway ID**	**Name**	***P* value**
rno01100	Metabolic pathways	1.3E-3
rno05016	Huntington's disease	3.2E-3
rno01200	Carbon metabolism	1.4E-2
rno05010	Alzheimer's disease	3.1E-2
rno00640	Propanoate metabolism	4.2E-2
rno00620	Pyruvate metabolism	6.0E-2
rno00480	Glutathione metabolism	8.6E-2

### Module screening from the protein-protein interaction network

**Figure 3:**
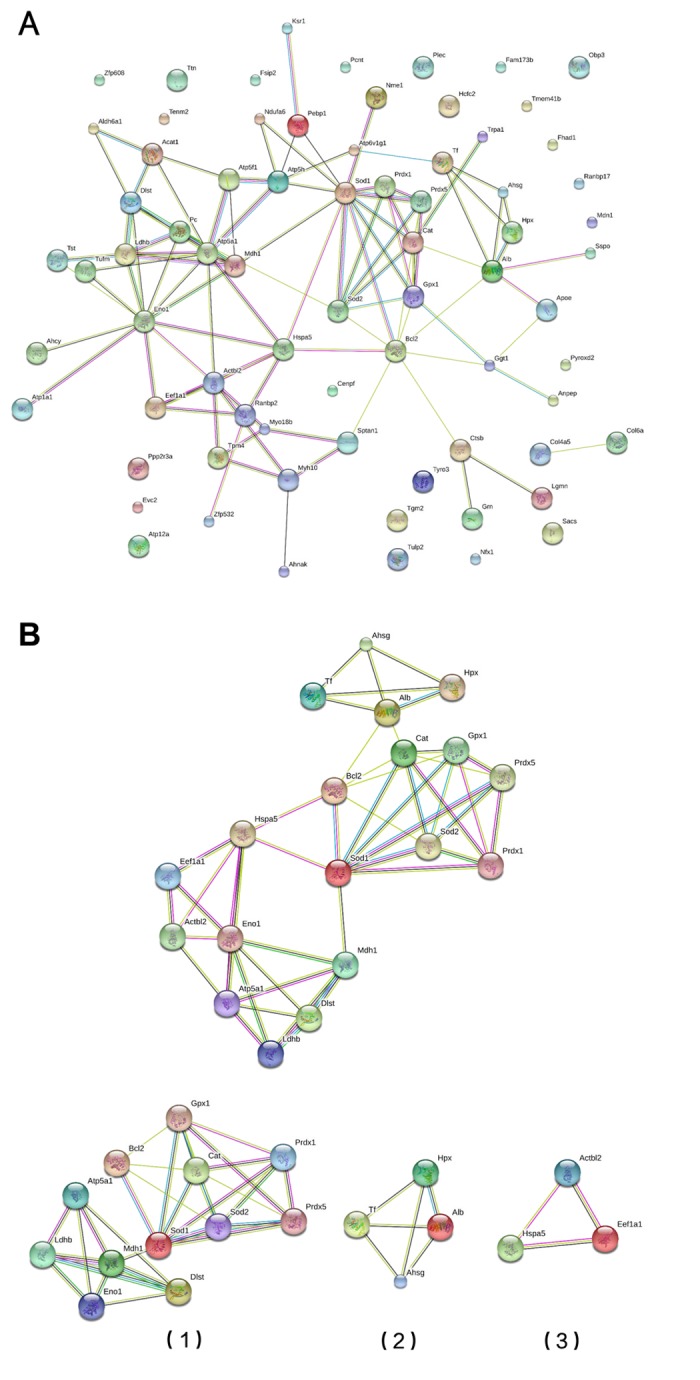
**Module screening of a PPI network of DEPs.** (**A**) Protein-protein interaction network of DEPs (STRING). (**B**) PPI network modules screened using the Molecular Complex Detection plug-in. Three PPI network modules were screened (1, 2 and 3).

A protein-protein interaction (PPI) network was constructed using the STRING database (Figure 3A). Using information from the database, we screened for the top 10 high-degree hub nodes. These hub proteins were SOD1, ENO1, BCL2, ATP5A1, SOD2, CAT, GPX1, ACTBL2, LCHB, and ATP5H. Among them, SOD1 was the highest-degree (12) node. Moreover, a total of 73 nodes and 99 edges were analyzed using the MCODE plug-in. The top 3 significant modules were selected (Figure 3B), and the functional annotation of the genes involved in the modules was analyzed. Enrichment analysis of most of the proteins in modules 1-3 is shown in Table 4. GO molecular function analysis showed that DEPs were mainly involved in oxidative stress, while the GO cellular component analysis showed that DEPs were mainly located in the mitochondria, and the KEGG analysis showed that the genes were mainly associated with the peroxisome pathway.

**Table 4:** Gene ontology/ KEGG pathway analysis of the top 3 significant modules.

**Biological Process (GO)**				
**#pathway ID**	**pathway description**	**Count**	***P* value**	**Matching proteins**
GO:0010035	response to inorganic substance	8	2.73E-06	Alb,Bcl2,Cat,Gpx1,Hspa5,Sod1,Sod2,Tf
GO:0042743	hydrogen peroxide metabolic process	4	2.73E-06	Cat,Gpx1,Sod1,Sod2
GO:0010038	response to metal ion	7	5.50E-06	Alb,Bcl2,Cat,Hspa5,Sod1,Sod2,Tf
GO:1901564	organonitrogen compound metabolic process	9	5.50E-06	Dlst,Eef1a1,Eno1,Gpx1,Hpx,Ldhb,Mdh1,Sod1,Sod2
GO:0010033	response to organic substance	10	4.45E-05	Ahsg,Alb,Bcl2,Cat,Eef1a1,Gpx1,Hspa5,Sod1,Sod2,Tf
GO:0031667	response to nutrient levels	7	4.45E-05	Alb,Bcl2,Cat,Gpx1,Hspa5,Sod1,Sod2
GO:0006091	generation of precursor metabolites and energy	5	6.63E-05	Cat,Dlst,Eno1,Mdh1,Sod2
GO:1990267	response to transition metal nanoparticle	5	8.70E-05	Alb,Bcl2,Hspa5,Sod1,Sod2
GO:0055114	oxidation-reduction process	7	9.71E-05	Dlst,Gpx1,Ldhb,Mdh1,Prdx1,Prdx5,Sod1
GO:0033591	response to L-ascorbic acid	3	0.000148	Bcl2,Cat,Sod2
GO:0045333	cellular respiration	4	0.000148	Cat,Dlst,Mdh1,Sod2
GO:0046496	nicotinamide nucleotide metabolic process	4	0.000148	Dlst,Eno1,Ldhb,Mdh1
GO:0051186	cofactor metabolic process	5	0.00016	Dlst,Eno1,Hpx,Ldhb,Mdh1
GO:0043066	negative regulation of apoptotic process	6	0.000234	Alb,Bcl2,Cat,Hspa5,Sod1,Sod2
GO:0051881	regulation of mitochondrial membrane potential	3	0.000234	Bcl2,Sod1,Sod2
GO:0007584	response to nutrient	5	0.000438	Alb,Bcl2,Cat,Gpx1,Sod2
GO:0019674	NAD metabolic process	3	0.000458	Dlst,Ldhb,Mdh1
GO:0042311	vasodilation	3	0.000458	Alb,Sod1,Sod2
GO:0006749	glutathione metabolic process	3	0.00048	Gpx1,Sod1,Sod2
GO:0007568	aging	5	0.00064	Bcl2,Cat,Gpx1,Sod1,Sod2
GO:0071310	cellular response to organic substance	7	0.000785	Ahsg,Cat,Eef1a1,Hspa5,Sod1,Sod2,Tf
GO:0009060	aerobic respiration	3	0.000925	Cat,Dlst,Mdh1
GO:0033273	response to vitamin	4	0.000955	Bcl2,Cat,Gpx1,Sod2
GO:0044710	single-organism metabolic process	9	0.000955	Dlst,Eno1,Gpx1,Hpx,Ldhb,Mdh1,Prdx1,Prdx5,Sod1
GO:0055072	iron ion homeostasis	3	0.0011	Sod1,Sod2,Tf
GO:0006790	sulfur compound metabolic process	4	0.00113	Dlst,Gpx1,Sod1,Sod2
GO:0046686	response to cadmium ion	3	0.00113	Cat,Sod1,Sod2
GO:0006950	response to stress	8	0.00148	Ahsg,Alb,Bcl2,Cat,Gpx1,Prdx1,Sod1,Tf
GO:1901700	response to oxygen-containing compound	7	0.00164	Ahsg,Bcl2,Cat,Gpx1,Sod1,Sod2,Tf
GO:0034284	response to monosaccharide	4	0.00175	Bcl2,Cat,Gpx1,Sod2
GO:0045471	response to ethanol	4	0.002	Bcl2,Cat,Sod1,Sod2
GO:0010243	response to organonitrogen compound	6	0.00216	Ahsg,Bcl2,Cat,Gpx1,Sod1,Tf
GO:0009719	response to endogenous stimulus	7	0.00223	Ahsg,Bcl2,Cat,Eef1a1,Gpx1,Sod1,Tf
GO:0020027	hemoglobin metabolic process	2	0.00241	Cat,Hpx
GO:0050665	hydrogen peroxide biosynthetic process	2	0.00241	Sod1,Sod2
GO:0097305	response to alcohol	5	0.00246	Bcl2,Cat,Gpx1,Sod1,Sod2
GO:0009056	catabolic process	6	0.00316	Cat,Dlst,Eno1,Gpx1,Hspa5,Sod1
GO:0048583	regulation of response to stimulus	7	0.00343	Ahsg,Alb,Bcl2,Cat,Hpx,Hspa5,Mdh1
GO:0019430	removal of superoxide radicals	2	0.00369	Sod1,Sod2
GO:0042554	superoxide anion generation	2	0.00369	Sod1,Sod2
GO:0042744	hydrogen peroxide catabolic process	2	0.00369	Cat,Gpx1
GO:0006734	NADH metabolic process	2	0.00451	Dlst,Mdh1
GO:0034641	cellular nitrogen compound metabolic process	8	0.00451	Dlst,Eef1a1,Eno1,Gpx1,Ldhb,Mdh1,Sod1,Sod2
GO:0042221	response to chemical	8	0.00508	Ahsg,Alb,Bcl2,Cat,Eef1a1,Gpx1,Sod1,Tf
GO:0071451	cellular response to superoxide	2	0.00519	Sod1,Sod2
GO:0006979	response to oxidative stress	4	0.00595	Cat,Gpx1,Prdx1,Sod1
GO:0006518	peptide metabolic process	4	0.00647	Eef1a1,Gpx1,Sod1,Sod2
GO:0001836	release of cytochrome c from mitochondria	2	0.00695	Bcl2,Sod2
GO:0001666	response to hypoxia	4	0.00696	Bcl2,Cat,Sod2,Tf
GO:0001101	response to acid chemical	4	0.00709	Bcl2,Cat,Gpx1,Sod2
GO:0051593	response to folic acid	2	0.00894	Bcl2,Gpx1
**Molecular Function (GO)**				
GO:0016209	antioxidant activity	6	3.58E-09	Cat,Gpx1,Prdx1,Prdx5,Sod1,Sod2
GO:0016491	oxidoreductase activity	8	1.09E-06	Cat,Gpx1,Ldhb,Mdh1,Prdx1,Prdx5,Sod1,Sod2
GO:0004601	peroxidase activity	4	2.46E-06	Cat,Gpx1,Prdx1,Prdx5
GO:0005488	binding	14	0.0001	Alb,Atp5a1,Bcl2,Cat,Dlst,Eef1a1,Gpx1,Hpx,Ldhb,Mdh1,Prdx1,Sod1,Sod2,Tf
GO:0005515	protein binding	10	0.000409	Alb,Bcl2,Dlst,Eno1,Hspa5,Ldhb,Prdx1,Sod1,Sod2,Tf
GO:0043167	ion binding	11	0.000409	Alb,Atp5a1,Cat,Eef1a1,Eno1,Gpx1,Hpx,Hspa5,Sod1,Sod2,Tf
GO:1901363	heterocyclic compound binding	10	0.000558	Alb,Atp5a1,Cat,Eef1a1,Hspa5,Ldhb,Mdh1,Prdx1,Sod2,Tf
GO:0097159	organic cyclic compound binding	10	0.000583	Alb,Atp5a1,Cat,Eef1a1,Hspa5,Ldhb,Mdh1,Prdx1,Sod2,Tf
GO:0042802	identical protein binding	5	0.0013	Bcl2,Eno1,Ldhb,Prdx1,Sod2
GO:0036094	small molecule binding	8	0.00136	Alb,Atp5a1,Cat,Eef1a1,Hspa5,Ldhb,Mdh1,Tf
GO:0003824	catalytic activity	10	0.00164	Dlst,Eef1a1,Eno1,Gpx1,Ldhb,Mdh1,Prdx1,Prdx5,Sod1,Sod2
GO:0004784	superoxide dismutase activity	2	0.00217	Sod1,Sod2
GO:0051920	peroxiredoxin activity	2	0.00217	Prdx1,Prdx5
GO:0048037	cofactor binding	4	0.00259	Alb,Cat,Ldhb,Mdh1
GO:0000166	nucleotide binding	7	0.0039	Atp5a1,Cat,Eef1a1,Hspa5,Ldhb,Mdh1,Tf

**Cellular Component (GO)**				
GO:0005739	mitochondrion	10	2.22E-07	Atp5a1,Bcl2,Cat,Dlst,Hspa5,Ldhb,Mdh1,Prdx1,Prdx5,Sod1
GO:0043209	myelin sheath	5	1.86E-05	Hspa5,Ldhb,Mdh1,Sod1,Sod2
GO:0005615	extracellular space	7	9.17E-05	Ahsg,Alb,Cat,Hpx,Mdh1,Sod1,Tf
GO:0005740	mitochondrial envelope	5	0.00154	Atp5a1,Bcl2,Cat,Sod1,Sod2
GO:0031012	extracellular matrix	4	0.00154	Ahsg,Alb,Sod1,Tf
GO:0032991	macromolecular complex	9	0.00154	Ahsg,Alb,Atp5a1,Dlst,Eno1,Hspa5,Prdx1,Sod1,Tf
GO:0044444	cytoplasmic part	11	0.00154	Atp5a1,Bcl2,Dlst,Eno1,Gpx1,Ldhb,Mdh1,Prdx1,Prdx5,Sod1,Tf
GO:0043227	membrane-bounded organelle	12	0.00171	Atp5a1,Bcl2,Dlst,Eef1a1,Gpx1,Hpx,Ldhb,Mdh1,Prdx1,Prdx5,Sod1,Tf
GO:0043234	protein complex	8	0.00239	Ahsg,Alb,Atp5a1,Dlst,Eno1,Hspa5,Sod1,Tf
GO:0044429	mitochondrial part	5	0.00239	Atp5a1,Bcl2,Cat,Prdx1,Sod1
GO:0070062	extracellular exosome	6	0.00239	Cat,Hpx,Hspa5,Ldhb,Mdh1,Sod1
GO:0044421	extracellular region part	7	0.00262	Ahsg,Alb,Hpx,Hspa5,Ldhb,Mdh1,Tf
GO:0005777	peroxisome	3	0.00322	Prdx1,Prdx5,Sod1
GO:0043231	intracellular membrane-bounded organelle	11	0.00334	Atp5a1,Bcl2,Dlst,Eef1a1,Gpx1,Ldhb,Mdh1,Prdx1,Prdx5,Sod1,Tf
**KEGG Pathways**				
4146	Peroxisome	5	1.35E-06	Cat,Prdx1,Prdx5,Sod1,Sod2
5014	Amyotrophic lateral sclerosis (ALS)	4	1.16E-05	Bcl2,Cat,Gpx1,Sod1
1120	Microbial metabolism in diverse environments	4	0.000697	Cat,Dlst,Ldhb,Mdh1
5016	Huntington s disease	4	0.001	Atp5a1,Gpx1,Sod1,Sod2

### SOD1 and the Ac-NF-κB signaling pathway in renal aging

**Figure 4:**
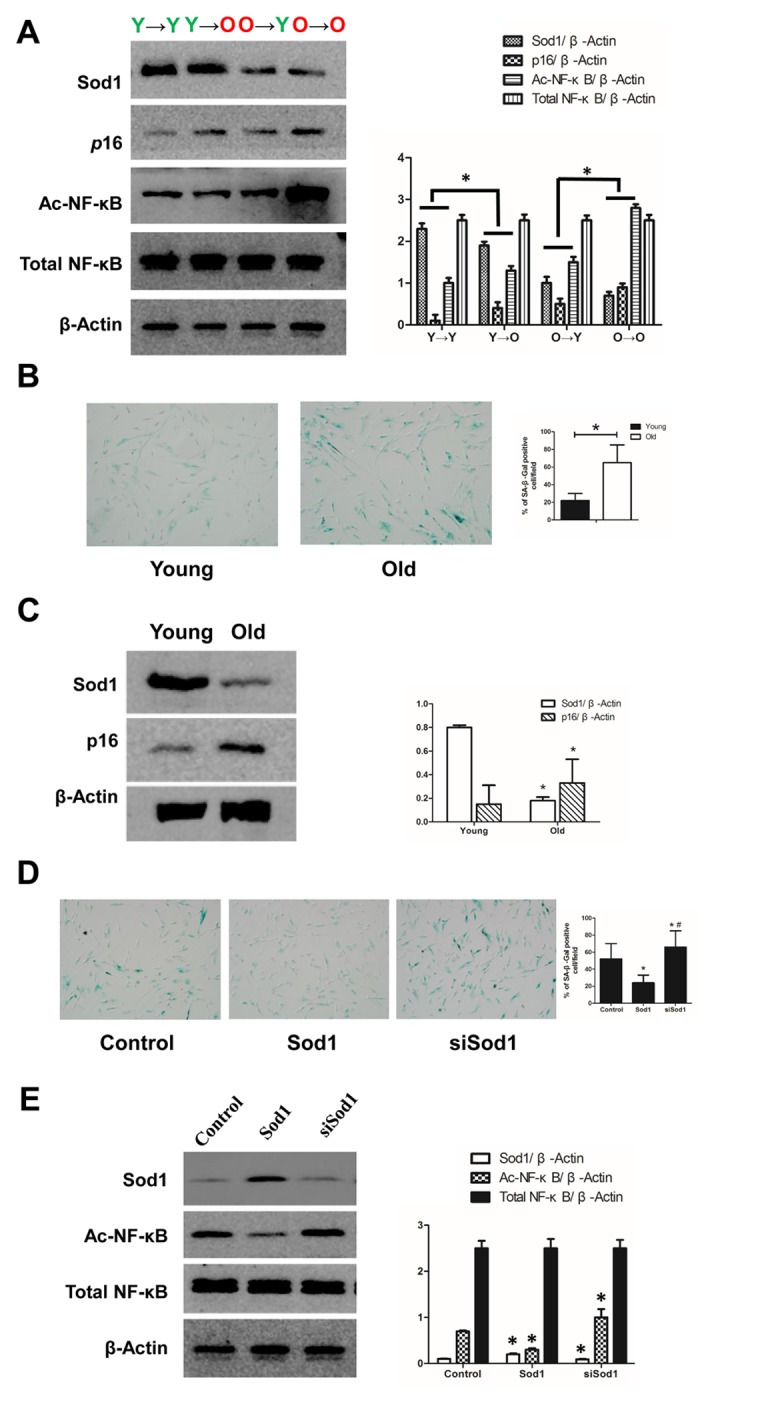
**Expression of SOD1 and NF-κB *in vivo* and *in vitro.*** (**A**) Expression of SOD1, p16 and NF-κB in kidney tissue from each group (*p < 0.05). (**B**) SA-β-gal staining of young and old tubular epithelial cells. (**C**) Expression of SOD1 and p16 in young and old tubular epithelial cells (*p < 0.05 vs young group). (**D**) SA-β-gal staining of tubular epithelial cells after the indicated interventions (*p < 0.05 vs control; #p < 0.05 vs SOD1 overexpression). (**D**) Expression of NF-κB in tubular epithelial cells after the indicated interventions (*p < 0.05 vs control).

Because SOD1 was the highest-degree node in the PPI network of DEPs, we verified its expression *in vivo.* We found that SOD1 expression was lower in the Y→O than the Y→Y group, and was higher in the O→Y than the O→O group. In addition, we observed that acetylated (Ac)-NF-κB showed the opposite expression profile (Figure 4A). *In vitro.* Sod1 was broadly expressed in renal cells, especially in tubular epithelial cells. When renal tubular epithelial cells isolated from the young and old rats were cultured in the same culture medium under the same culture conditions, Sod1 expression was lower in aged tubular epithelial cells (Figure 4C). This finding was consistent with our earlier proteomics results summarized above.

We also observed that Sod1 knockdown led to increased Ac-NF-κB expression and that overexpression of SOD1 had the opposite effect. In both cases, total NF-κB was unchanged. (Figure 4D). Thus, Sod1 knockdown appears to promote cell senescence, while Sod1 overexpression appears to inhibit it.

## DISCUSSION

This study had three main aspects. First, we successfully established a young-old rat kidney transplant model. Second, we found that the local renal environment could affect renal aging and might make the kidney younger/older by affecting oxidative stress/mitochondria function. Third, we determined that Sod1, detected in a proteomic analysis, inhibits renal aging.

The incidence of morbidity associated with chronic kidney disease related to aging has increased significantly. Renal aging primarily manifests as declining glomerular GFR, impaired sodium balance in the renal tubules, impaired fluid balance, potassium retention, declining urine dilution, decreased ability to reduce urinary pH, decreased effective renal plasma flow, increased filtration fraction, increasing glomerular vascular resistance, impaired vasodilator response, and endocrine-associated changes, including decreased plasma renin and aldosterone, decreased response of EPO to anemia, and decreased vitamin D activity [5,6]. Multiple factors ranging from genetic background to chronic inflammation contribute to renal aging [7]. For example, regulatory genes and post-transcriptional processes (acetylation and methylation) are essential for regulation of renal cell differentiation and maintenance of cellular function [8]. In the normal aging rat, a variety of proteins are expressed in the glomeruli of the elderly but not young rats. These include proteins within pathways that mediate glomerular sclerosis and interstitial fibrosis, thereby accelerating renal aging [9]. In addition, through functional genomics more than 500 genes have been found to be differentially expressed between neonates (8 weeks) and the elderly (88 years). Proteins overexpressed in the elderly kidney were involved in immune responses, inflammation, extracellular matrix synthesis, oxidation processes, glycolipid metabolism and collagen degradation [10].

To observe the effect of the local renal environment on renal aging, we established a young-old rat kidney transplant model. To exclude effects of renal ischemia and reperfusion factors, we included Y→Y and O→O kidney transplant control groups. To exclude effects of rejection factors, we established the model using inbred F344 rats. This rat renal allograft transplantation model has few complications and a good survival rate, providing a practical and reliable model for further experimentation. Importantly, there were no significant differences in renal function or histomorphology between the O→O and O→Y transplanted kidneys or between the Y→Y and Y→O transplanted kidneys, which further suggests the model was successfully established.

Our GO and KEGG analyses suggest the local renal environment can indeed affect aging of the kidney. Construction of a PPI network using the STRING database revealed SOD1, ENO1, BCL2, ATP5A1, SOD2, CAT, GPX1, ACTBL2, LCHB and ATP5H to have high degrees of connectivity, and SOD1 to have the highest. Notably, SOD1 suppressed renal cell aging in this study by inhibiting NF-κB. SOD1 is the predominant form of SOD in the cytoplasm, where it acts as an antioxidant [11]. Results from invertebrates also suggest a role for SOD1 in aging. Overexpression of SOD1 in short-lived strains of fruit files extends their lifespan and appears to delay aging [12]. The formation of free radicals increases with age, and SOD1 is an important factor reducing oxidative stress [13]. Ac-NF-κB reportedly contributes to the process of cell senescence by acting in the nucleus to stimulate transcription of senescence-related genes. SOD1 may inhibit senescence by reducing the level of Ac-NF-κB [14]. Consistent with that idea, we observed that SOD1 knockdown led to increased levels of Ac-NF-κB.

Among the other screened hub proteins, ENO1, is reportedly involved in aging and age-related diseases, such as Alzheimer’s disease [15,16], while BCL2, belongs to an anti-apoptotic protein family. Expression of BCL2 is reduced during aging and so could be a target for anti-aging therapy [17]. ATP5A1 and SOD2 are reportedly involved in mitochondrial dysfunction [18], and CAT expression is decreased in the hearts of aged rats. In addition to contributing to H_2_O_2_ detoxification, CAT protects against NO/peroxynitrite and may be involved in regulating angiogenesis, neovascularization and apoptosis [19]. GPX1 is also involved in the aging process, and its overexpression in aged mice suppresses arterial and venous thrombosis [20]. ATP5H is related to ATP synthase function, and the ATP5H/KCTD2 locus is associated with age-related Alzheimer’s disease risk [21]. A total of 73 nodes and 99 edges were analyzed using the MCODE plug-in, and 3 significant modules were selected. It is noteworthy that the Go molecular function analysis showed that DEPs were mainly involved in oxidative stress, the GO cellular component analysis showed they mainly localize to mitochondria, and the KEGG analysis showed they mainly associate with the peroxisome pathway. We therefore suggest renal aging reflects the local renal environment, particularly oxidative stress and mitochondrial function.

In summary, our findings indicate a young-old rat kidney transplant model can provide a basis for studying the effects of the local renal environment on renal aging and indicates that the status of the local renal environment, particularly oxidative stress/mitochondrial function, is a key determinant of kidney aging.

## MATERIALS AND METHODS

### Animals

Male F344 rats were purchased from the Si Bei Fu Laboratory Animal Company (Beijing, China). Rats were housed under specific pathogen-free conditions in the Experimental Animal Center of the Chinese General Hospital of PLA: 22 ± 1°C, 40% humidity, 12/12-h light/dark cycle, with free access to water. Young (3 months) and old (20 months) rats were used in this study. As shown in Figure 1A, the rats were divided into six groups: young and old controls and kidney transplanted from young rat to young rat (Y→Y), from young rat to old rat (Y→O), from old rat to young rat (O→Y), and from old rat to old rat (O→O). Rats were sacrificed 16 weeks after transplantation, and the transplanted kidney was further studied. All experimental protocols were approved by the Animal Care Committee of Chinese General Hospital of PLA.

### Establishment of the model

Rats were anesthetized using isoflurane (5%, induction of anesthesia; 2.2% 0.3-0.6 L/min, during surgery; the concentration was adjusted according to the depth of anesthesia). Transplantation was performed with one kidney. After separating the renal vessels, the rats were heparinized (500 U/ml, 2 ml), and a renal protection fluid was used for hypothermic irrigation of the transplanted kidney. The transplanted kidney, vessels, ureter and bladder disc were then excised from the donor rat, after which the kidney was transplanted to the donee through vascular and urinary reconstruction. A rat might be both a donor and a donee. After surgery, the rats were placed on an insulation blanket and were intraperitoneally administered penicillin (400,000 U).

### Histopathological analysis

Kidney slices were fixed in 10% formalin solution overnight. After automated dehydration through a graded alcohol series, transverse kidney slices were embedded in paraffin, sectioned at 4 μm, and stained with hematoxylin-eosin and periodic acid-Schiff (PAS). Histological examinations were performed independently in a blinded fashion by two observers. Quantitative analyses of kidney tissue were done using Image-Pro software (Media Cybernetics Inc., Silver Springs, MD, USA) with 20 randomly selected ×200 fields per rat.

### Senescence-associated β-galactosidase staining

Cryostat sections (4 μm) were mounted on glass slides and fixed in 0.2% glutaraldehyde and 2% formaldehyde at room temperature for 15 min. The sections were then washed in PBS, incubated in freshly prepared senescence-associated β-galactosidase (SA-β-gal) staining solution overnight at 37°C, counterstained with eosin, and examined under a microscope. An investigator blinded to the sample identity performed the image analysis. Quantitative analysis of SA-β-gal positive-stained areas was performed using Image-Pro software with 20 randomly selected ×200 fields per rat.

### Western blot analysis

Protein concentrations were determined using a Pierce BCA assay kit (Thermo Fisher Scientific, Waltham, MA, USA). Aliquots of protein (50-100 μg) were separated with 6-16% SDS-PAGE, transferred to nitrocellulose membranes, blocked with blocking buffer for 1 h at room temperature, and incubated with primary antibodies at 4°C overnight. Blots were subsequently incubated with secondary immunoglobulins conjugated with horseradish peroxidase. Immunoreactive bands were visualized using enhanced chemiluminescence, and densitometry was performed using Quantity One software (Bio-Rad Laboratories, Hercules, CA, USA). Band intensities were quantified using ImageJ software (NIH, Bethesda, MD, USA).

### Sample collection and protein extraction

Kidney sample collection and protein extraction were performed according to a standard procedure [22].

### Enzyme-linked immunosorbent assay (ELISA)

After collecting tissue samples from the transplanted kidneys, TNF-α and IL-1β levels were determined by using ELISA kits (R&D Systems Inc, Minneapolis, MN) according to the manufacturer’s instructions. Levels of the cytokines were normalized to the protein concentration in the lysate.

### LC-MS/MS analysis

Digested peptide mixtures were pressure-loaded onto a fused silica capillary column packed with 3-μm Dionex C18 material (RP; Phenomenex). The RP sections with 100 Å were 15 cm long, and the column was washed with buffer A (water, 0.1% formic acid) and buffer B (acetonitrile, 0.1% formic acid). After desalting, a 5-mm, 300-μm C18 capture tip was placed in line with an Agilent 1100 quaternary high-performance liquid chromatograph and analyzed using a 12-step separation. The first step consisted of a 5-min gradient from 0% to 2% buffer B, followed by a 45-min gradient to 40% buffer B. Next, buffer B flowed in a 3-min gradient from 40% to 80% and 10-min of 80% buffer B. After a 2-min buffer B gradient from 80% to 2%, approximately 100 µg of a tryptic peptide mixture was loaded onto the columns and was separated at a 0.5 µL/min ﬂow rate using a linear gradient. As peptides were eluted from the microcapillary column, they were electrosprayed directly into a micrOTOF-Q II mass spectrometer (BRUKER Scientiﬁc) with application of a distal 180°C temperature source. The mass spectrometer was operated in the MS/MS (auto) mode. Survey MS scans were acquired in the TOF-Q II with the resolution set to a value of 20,000. Each survey scan (50-2,500) was followed by five data-dependent tandem mass (MS/MS) scans at a 2 Hz normalized scan speed. Tandem mass spectra were searched against the mascot 2.1 (Local Host) RAT protein database. The search results were then filtered using a cutoff of 1% for a peptide false identification rate. Peptides with a Z score < 4 or Delta-Mass > 5 ppm were rejected. Furthermore, the minimum number of peptides used to identify a protein was set to 1. The default parameters for the Profile Analysis 2.0 software were used throughout the analysis.

### Bioinformatics analysis

To analyze DEPs at a functional level, gene ontology (GO) enrichment and KEGG pathway analyses were performed using the DAVID online tool (https://david.ncifcrf.gov/). To evaluate the interactive relationships among DEPs, we mapped them to STRING (www.string-db.org/) and experimentally validated the interactions. Those with a combined score > 0.4 were selected as significant. PPI networks were constructed using Cytoscape software. The plug-in Molecular Complex Detection (MCODE) was used to screen the modules of the PPI network in Cytoscape. The criteria were set as follows: MCODE scores > 3 and number of nodes > 4.

### Cell culture and transfections

Isolation and culture of rat primary tubular epithelial cells were performed as described previously [23]. Young and aging renal tubular epithelial cells were respectively isolated from young and old rat kidneys and cultured in RPMI 1640 medium supplemented with 10% fetal bovine serum. After three passages, the cells were collected for experimentation. Small interfering RNAs (siRNAs) targeting Sod1, which is highly expressed in tubular epithelial cells, and a RNAi negative control duplex were purchased from Santa Cruz Biotechnology. The RNAi oligonucleotide and RNAi negative control duplex were transfected into cells as instructed by the manufacturer.

### Statistical analysis

All data analyses were performed using SPSS software (ver. 18.0; SPSS, Chicago, IL, USA). Data are expressed as the mean ± SD. Comparisons among groups were made using analysis of variance. Values of *p* < 0.05 were considered significant.
